# Performance of a Simple Energetic-Converting Reaction Model Using Linear Irreversible Thermodynamics

**DOI:** 10.3390/e21111030

**Published:** 2019-10-24

**Authors:** J. C. Chimal-Eguia, R. Paez-Hernandez, Delfino Ladino-Luna, Juan Manuel Velázquez-Arcos

**Affiliations:** 1 Centro de Investigación en Computación del Instituto Politécnico Nacional, Av. Miguel Othon de Mendizabal s/n. Col. La Escalera, Ciudad de México, CP 07738, Mexico; 2 Área de Física de Procesos Irreversibles, Departamento de Ciencias Básicas, Universidad Autónoma Metropolitana, U-Azcapotzalco, Av. San Pablo 180, Col.Reynosa, Ciudad de México, CP 02200, Mexico; rpaez.uam@gmail.com (R.P.-H.); dll@correo.azc.uam.mx (D.L.-L.); jmva@correo.azc.uam.mx (J.M.V.-A.)

**Keywords:** linear irreversible thermodynamics, maximum power output, maximum ecological Function, maximum efficient power function, enzymatic reaction model

## Abstract

In this paper, the methodology of the so-called Linear Irreversible Thermodynamics (LIT) is applied to analyze the properties of an energetic-converting biological process using simple model for an enzymatic reaction that couples one exothermic and one endothermic reaction in the same fashion as Diaz-Hernandez et al. (*Physica A*, **2010**, *389*, 3476–3483). We extend the former analysis to consider three different operating regimes; namely, Maximum Power Output (MPO), Maximum Ecological Function (MEF) and Maximum Efficient Power Function (MEPF), respectively. Based on the later, it is possible to generalize the obtained results. Additionally, results show analogies in the optimal performance between the different optimization criteria where all thermodynamic features are determined by three parameters (the chemical potential gap $\Delta =\frac{\mu_{1}-\mu_{4}}{RT}$, the degree of coupling *q* and the efficiency η). This depends on the election that leads to more or less efficient energy exchange.

## 1. Introduction

A very interesting problem in non-equilibrium thermodynamics and in the theory of thermodynamics in general, is to determine the efficiency with which energy is exchanged. In fact, in many biological systems, the transfer of energy is of decisive importance. It is well known that all intracellular processes can be studied as chemical reactions of some kind, and that many of the biochemical reactions in living organisms have been seen to be catalyzed by enzymes; there are some good examples where the energetic properties studied are really relevant [1].

Considering the classical ideas of thermodynamics when one wants to analyze biological systems, it is typical to take the free energy of the biological system and convert it into work. For instance, to carry out a transport process or a chemical reaction, it is usual for this type of study to focus on analyzing the energetic properties of such systems. Note, however, that the subject is hard to study from the classical perspective of thermodynamics, since the temperature in many biological systems is homogeneous [2]).

An additional step to analyze the energetic properties of a simple energy converter was given by Curzon and Ahlborn in 1975 [3], who proposed a model which operates between two heat sources with high and low temperatures, $T_{h}$ and $T_{c}$$\left(T_{c}<T_{h}\right)$, respectively. They found an expression for the efficiency at maximum power output given by $\eta_{CA}=1-\sqrt{T_{c}/T_{h}}$ , a result that in principle is independent of the model parameters and only depends on the temperatures of the heat reservoirs; analogously this is what happens with the efficiency for a reversible Carnot cycle $\eta_{C}=1-T_{c}/T_{h}$.

Considering the Curzon and Alhbor article, many authors began to introduce different objective functions, among others, such as the ecological function [4], the omega function [5], the efficient power function [6], etc. All of them trying to obtain efficiency and power values mainly for real power plants, but also heat pumps and refrigerators [7,8].

Moreover, it was reported [9,10,11] that thermal engines show some universality regarding the behavior of the efficiency when it works at the maximum power regime [11], although the analyzed models are different in nature and scale [12,13,14]. Recently, some thermal engines with kinetic [15,16,17] and mesoscopic [18] descriptions were published as examples of devices which convert non-thermal energy (mainly chemical energy) into useful work. The importance of these models is that the energy production processes seen in the molecular biological level obey similar principles as those observed in the classical thermal engines [19].

On the other hand, Kedem et al. [20] published in 1965 the first step of a non-equilibrium theory towards a description of linear converters of energy (which would be called Linear Irreversible Thermodynamics, LIT). Since then, many authors have agreed in considering this theory as a basis for the analysis of non-equilibrium systems, (particularly, in biological systems remarkably close to the equilibrium). One of the relevant questions tackled by Kedem et al. at that time was to answer which was the maximal efficiency of the oxidative phosphorylation in an isolated mitochondrion. Kandem et al. obtained some qualitative predictions confirmed by experimental data.

For biological process, for instance, several authors have studied different optimal regimes like Prigogine [21] with his minimum entropy production theorem. Odun and Pinkerton [22] who analyzed the maximum power output regime for various biological systems, Stucki [2] who introduced some optimal criteria to study the optimum oxidative phosphorylation regime, among others [23,24,25,26,27,28] who have studied many biological energy conversion processes by means of the LIT where some optimum performance regimes have been analyzed.

In this context, we have decided to study the thermodynamical properties of an energetic converting biological process, using for this purpose a simple model for an enzymatic reaction that couples one exothermic and one endothermic reaction in the same fashion described by Diaz-Hernandez et al. [15], but now using the Linear Irreversible Thermodynamics (LIT) for three different operating regimes,;namely, Maximum Power Output (MPO), Maximum Ecological Function (MEF) and Maximum Efficient Power Function (MEPF), respectively.

The paper is organized as follows: Section 2 introduces a model and the phenomenological flow equations of a remarkably simple system enzymatic reaction coupled with ATP hydrolysis. Section 3 presents the analysis of the optimal operation regimes in the context of the Linear Irreversible Thermodynamics. Finally, Section 4 gives some concluding remarks.

## 2. The Model

Consider a simple enzymatic reaction coupled with ATP hydrolysis which might be written as [15]:

(1) $$\begin{array}{c} E+X+ATP⇌\left[EX\right]+ADP⇌\left[EY\right]+P_{i}+ADP\\ ⇌E+Y+P_{i}+ADP \end{array}$$

where *E* represents the enzyme, *X* is the substrate and *Y* is the product. Besides [EX] and [EY] are transient complexes of the enzyme with the substrate and the product respectively, ATP corresponds with the Adenosine Triphosphate, ADP is the Adenosine Diphosphate and $P_{i}$ represents the inorganic phosphate.

Considering the first part of Equation (1), it is possible to obtain the respective reaction velocity, which, according to the mass action law, is given by:

(2) $$\frac{d[E]}{dt}=-k_{1}\left[E\right]\left[X\right]+k_{-1}\left[EX\right]$$

**Figure 1:**
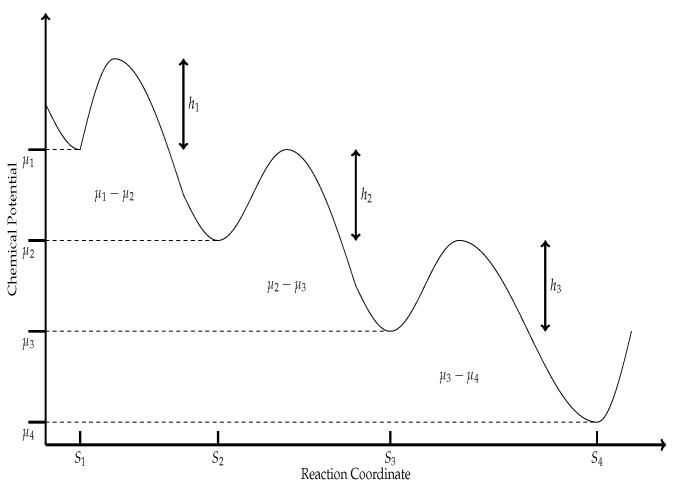
Chemical potential scheme necessary for the reactions in Equation (1) to proceed forward.

Now, using Arrhenius law, which establishes that,

(3) $$k_{1}=F_{1}e^{-E_{1}/RT}\;\mathrm{and}\;k_{-1}=F_{-1}e^{-E_{-1}/RT}$$

where, $F_{1}$ and $F_{-1}$ are the frequency factors, $E_{1}$ and $E_{-1}$ are the activation reaction energies usually expressed in $\frac{cal}{mol}$ and *R* is the gas constant (expressed in $JK^{-1}mol^{-1}$). Now, from Figure 1, it is clear that both activation energies can be expressed as:

(4) $$E_{1}=h_{1}+\mu_{2}-\mu_{1}\;\mathrm{and}\;E_{-1}=h_{1}$$

where $\mu_{i}$ (i=1,2,3,4) is the corresponding chemical potential to the *i*th state along with the reaction sequence, and $h_{i}$ (i=1,2,3) is the minimum energy required for a collision between molecules to result in a chemical reaction, see Figure 1.

Hence, using Equations (3) and (4) and substituting them into Equation (2), we obtain the net velocities for the three reactions in (1) as:

(5) $$w_{i}=\xi_{i}\left(e^{-h_{i}/RT}-e^{-(h_{i}+\mu_{i}-\mu_{i+1})/RT}\right)$$

where i=1,2,3 and $\xi_{i}$ is defined in terms of the molar concentrations and the frequency factors [29]. Then, after some algebra, the above equation can be written as,

(6) $$w_{i}=A_{i}\left(1-e^{-(\mu_{i}-\mu_{i+1})/RT}\right)$$

where $A_{i}=\xi_{i}e^{-h_{i}/RT}$ (with i=1,2,3) and $\xi_{i}$ is defined as before.

Therefore, by using Equation (6) we can obtain the respective velocities of each reaction as;

(7) $$w_{1}=A_{1}\left(1-e^{-(\mu_{1}-\mu_{2})/RT}\right);\;w_{2}=A_{2}\left(1-e^{-(\mu_{2}-\mu_{3})/RT}\right);\;w_{3}=A_{3}\left(1-e^{-(\mu_{3}-\mu_{4})/RT}\right)\;$$

Now, considering for simplicity that $A_{1}=A_{3}=A$ and defining $A_{2}=\beta A$ (later we will see that β is going to be related to the coupling coefficient *q*), as Diaz-Hernandez et. al. [15] did in their model using a different approach, we can re-write Equations (7) as;

(8) $$\begin{array}{c} w_{1}=A\left(1-e^{-(\mu_{1}-\mu_{2})/RT}\right)\\ w_{2}=A_{2}\left(1-e^{-(\mu_{2}-\mu_{3})/RT}\right)\\ w_{3}=A\left(1-e^{-(\mu_{3}-\mu_{4})/RT}\right) \end{array}$$

Since for reactions near equilibrium, the affinity is small, making a Taylor expansion around zero is justified. Keeping this in mind, it is possible to transform Equation (8) into;

(9) $$\begin{array}{c} w_{1}=A\left(\mu_{1}-\mu_{2}\right)/RT+O\left(2\right)\\ w_{2}=A_{2}\left(\mu_{2}-\mu_{3}\right)/RT+O\left(2\right)\\ w_{3}=A\left(\mu_{3}-\mu_{4}\right)/RT+O\left(2\right) \end{array}$$

where we just keep the linear terms in the expansion.

From classical non-equilibrium studies, we can, under suitable conditions, define macroscopic variables locally, as gradients and flux densities. Such variables are called “thermodynamic forces” which drives flux densities often called “fluxes”. Following the Onsager formalism [30] we can establish a relation between such forces and fluxes near the steady thermodynamically non-equilibrium regime naming them phenomenological relations [26], given by

(10) $$J_{\delta }=\sum_{\psi }L_{\delta \psi }X_{\psi }$$

where, $L_{\delta \psi }$ are the phenomenological coefficients usually depending on the intensive variables which describes the coupling between two irreversible process δ and ψ, and $X_{\psi }$ are the respective thermodynamic forces. It is worthwhile to mention that in 1931 Onsager [30] demonstrated that for a system of flows and forces based on an appropriate dissipation function, the matrix of coefficients is symmetrical so that the phenomenological coefficients have the following symmetry relation $L_{\delta \psi }=L_{\psi \delta }$, which affords a considerable reduction in the number of coefficients measured.

Then, taking the above into account, it is possible for our system, to establish two thermodynamic flows $J_{1}$ and $J_{2}$ for which, we may write the following phenomenological equations;

(11) $$\begin{array}{c} J_{1}=L_{11}X_{1}+L_{12}X_{2}\\ J_{2}=L_{21}X_{1}+L_{22}X_{2} \end{array}$$

where, we are assuming that $L_{12}=L_{21}$.

In the classical equations of chemical kinetics, which are known to describe a chemical process quite precisely, the reaction rates are proportional to the concentrations. On the other hand, phenomenological equations require that the reaction velocity are proportional to the thermodynamic force, which in this case is the Affinity, which is in turn proportional to logarithms of concentration. To remove this inconsistency, we must consider this phenomenological description in the neighborhood of equilibrium when the rate of chemical change is sufficiently slow [31].

According to earlier considerations, if we consider that the driving force for the reaction is the affinity, then close to equilibrium, the chemical flow $J_{chem}$ should be proportional to the force:

(12) $$J_{chem}=L_{ij}\alpha_{i}=L_{ij}\left(\mu_{i}-\mu_{j}\right)$$

where $L_{ij}$ are the phenomenological coefficients and $\alpha_{i}=\mu_{i}-\mu_{j}$ is the Affinity. Therefore, assuming that for our chemical reactions the phenomenological relation between fluxes and forces is,

(13) $$J_{1}=w_{1}+w_{2}+w_{3}$$

where $w_{i}$ (i=1,2,3) are the net velocities defined in Equation (9). Then, from Equation (13) and substituting Equation (9) in it, we can write;

(14) $$J_{1}=A\left(\mu_{1}-\mu_{2}\right)+A_{2}\left(\mu_{2}-\mu_{3}\right)+A\left(\mu_{3}-\mu_{4}\right)$$

which can be rewritten as:

(15) $$J_{1}=A\left(\mu_{1}-\mu_{4}\right)+\left(A_{2}-A\right)\left(\mu_{2}-\mu_{3}\right)$$

It is worthwhile to analyze Equation (15). From the scheme in Figure 1, we observe that it corresponds to three sequential equations, all three reactions can be lumped into a single global reaction with free energy change for this reaction as $\Delta G_{TOT}=A\left(\mu_{1}-\mu_{4}\right)$. Of the three reactions represented in Figure 1, only in the second one, the consumed energy is used for an interesting purpose; the conversion of the substrate *X* into the product *Y*, and the free energy for this reaction is $\Delta G_{2}=A\left(\mu_{2}-\mu_{3}\right)$. So one possible physical meaning of $J_{1}$ is some dissipation-like energy. Then, we can later propose a linear flux–force relation for the enzymatic reaction model as,

(16) $$\begin{array}{c} J_{1}=A\left(\beta -1\right)\left(\mu_{2}-\mu_{3}\right)+A\left(\mu_{1}-\mu_{4}\right)\\ J_{2}=A\left(\mu_{2}-\mu_{3}\right)+A\left(\beta -1\right)\left(\mu_{1}-\mu_{4}\right) \end{array}$$

where in the context of linear irreversible thermodynamics we can identify $X_{1}=\mu_{2}-\mu_{3}$, $X_{2}=\mu_{1}-\mu_{4}$, $L_{12}=L_{21}=A$, and $L_{11}=L_{22}=A\left(\beta -1\right)$, where the parameter β was previously introduced in Equation (7).

We should note that $A_{i}$ is related to the minimum energy required for a collision between molecules. Thus, this energy could be different for the different stages in the enzymatic reaction causing the phenomenological coefficients $L_{ij}$ to be different, then the degree of coupling *q* is also different in each stage influencing the thermodynamic properties of the system (for instance, the power output or the entropy production). Considering the latter, we assume that the simplest case is one in which the coefficients $A_{i}$ are proportional to each other.

Following the concepts of classical thermodynamics, the efficiency function can be defined as [24],

(17) $$\eta =\frac{output}{input}=-\frac{J_{1}X_{1}}{J_{2}X_{2}}$$

From Equation (16), it is possible to substitute $J_{1}$ and $J_{2}$ into Equation (17), which yields,

(18) $$\eta =\frac{-x(q+Zx)}{qx+1/Z}$$

where,

$\;x=\frac{X_{1}}{X_{2}}=\frac{\mu_{2}-\mu_{3}}{\mu_{1}-\mu_{4}}$ is the stoichiometric coefficient,

$\;Z=\sqrt{\frac{L_{11}}{L_{22}}}=1$ is the phenomenological stoichiometry [2] and

$\;q=\frac{L_{12}}{\sqrt{L_{11}L_{22}}}=\frac{1}{\beta -1}$ is the degree of coupling.

Substituting these last expressions in Equation (18) we obtain,

(19) $$\eta =\frac{-x(1+(\beta -1)x)}{x+\beta -1}$$

**Figure 2:**
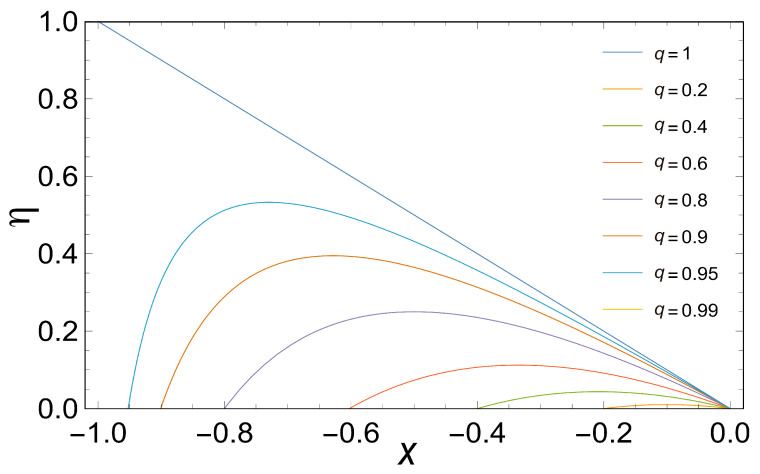
Dependence of the efficiency η on the stoichiometric coefficient *x* for different values of *q* from 0.2 up to 1. It is important to remember that the parameter β is related to the degree of coupling as $q=\frac{L_{12}}{\sqrt{L_{11}L_{22}}}=\frac{1}{\beta -1}$.

If we take the special case of complete coupling, i.e., q=1 (from Equation (18) we can notice that for q=1, β=2) in Equation (19), it is easy to observe that,

(20) $$\eta =-x=-\frac{X_{1}}{X_{2}}=\frac{\mu_{2}-\mu_{3}}{\mu_{1}-\mu_{4}}$$

which is highly similar to that obtained by Diaz-Hernandez et. al. [15] for a similar model using a different approach. Furthermore, Figure 2 shows the efficiency plotted as a function of the stoichiometric coefficient for various values of *q*. Note the fast decay of η with the decreasing of *q*.

## 3. Optimal Operation Regimes in the Context of the Linear Irreversible Thermodynamics

A very interesting problem in many biological systems is the transfer of energy which is of decisive importance ([23,24,26,27,28,32]). Caplan et al. [24] studied linear energy converters working in steady states, where they introduced definitions of power output and efficiency, besides the known notion of entropy production rate. Using the definitions of Caplan et al. of power output and efficiency, Stucki [2] analyzed some optimum regimes different from that of minimum entropy production studied before by Prigogine [33]. It has been of special interest in many systems (physical, chemical, biological, etc.) the study of some optimum working regimes for linear energy converters as a manner to understand the diverse ways in which the energy could be transferred [28]. So, let us analyze some of the most representative regimes found in the literature for the present system.

### 3.1. Maximum Power Output

Using the definitions of Caplan et al. [24] for linear energy converters it is possible to obtain the power output, working in a steady state at constant pressure and temperature, as following;

(21) $$P=-TJ_{1}X_{1}$$

Taking into account Equation (16) it is possible to substitute them into Equation (21), then we get,

(22) $$P=TL_{22}X_{2}^{2}q^{2}v\left(1-v\right)$$

where *v* is defined as $v=(-L_{11}/L_{12})x$, *q* and *x* are defined as in Equation (18) and *T* is the temperature.

Now, from Equation (19) it is possible to obtain *x* as a function of η as,

(23) $$x=\frac{-(1+\eta )\pm R}{2(\beta -1)}$$

with $R=\sqrt{{\left(1+\eta \right)}^{2}-4\eta {\left(\beta -1\right)}^{2}}$. Where, we also have considered that Z=1, using the definition given in Equation (18).

If we substitute Equation (23) in Equation (22) we obtain,

(24) $$P=\frac{\Delta^{2}TA}{2(\beta -1)}\left[\left(1+\eta \right)\pm R\right]\left[\frac{-(1+\eta \pm R)}{2}+1\right]$$

where $\Delta =\mu_{1}-\mu_{4}=X_{2}$, and *A* defined as in Equation (6).

It is important to notice that when we take q=1 in Equation (24), we obtain:

(25) P=ATΔη[Δ(1−η)]

which is very similar to the linear approximation of Equation (9) reported by Diaz-Hernandez et al. in Reference [15].

### 3.2. Maximum Ecological Function

Now, we are going to analyze a regime named ecological. In the context of the Finite Time Thermodynamics [4], the ecological function is defined as,

(26) E=P−Tσ

where *P* is the power output and σ the total entropy production (system plus surroundings) and T the temperature of the cold reservoir. However, in the context of the linear irreversible thermodynamics the ecological function takes the form [27]:

(27) $$E=-TL_{22}X_{2}^{2}\left(2x^{2}+3xq+1\right)$$

where again *q* and *x* are defined as in Equation (18) and *T* is the temperature.

Now, taking into account Equation (23) and substitute it into Equation (27) we obtain the ecological function as,

(28) $$\begin{array}{c} E=\frac{\Delta^{2}TA}{\left(1-\beta \right)}\{\left(-\eta \pm R\right)\left(\frac{-(1+\eta )\pm R}{2}+1\right)\\ +{\left(1-\beta \right)}^{2}-1\} \end{array}$$

with $R=\sqrt{{\left(1+\eta \right)}^{2}-4\eta {\left(1-\beta \right)}^{2}}$.

It is important to note that when we take q=1 in Equation (28), we obtain:

(29) E=ATΔ(2η−1)[Δ(1−η)]

which is remarkably similar to the linear approximation of Equation (10) reported by Diaz-Hernandez et al. in Reference [15].

**Figure 3:**
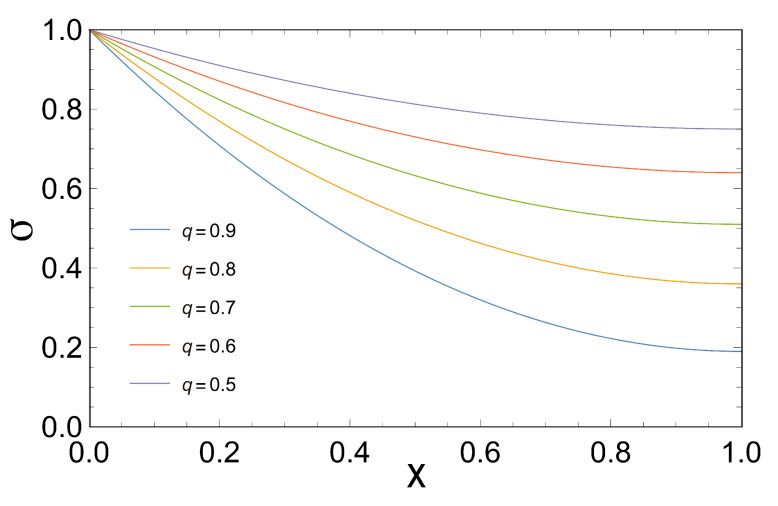
This Figure shows the entropy production σ versus the stoichiometric coefficient *x*. We observe that σ is a decreasing monotonous function with respect to x, for each value of *q*.

As can be seen in Figure 3, the entropy production is a decreasing monotonous function with respect to *x*, for each value of *q*. Besides, for a fixed *x*, we observe that when the entropy production grows *q* decreases. This could be important because, it seems that could exist a trade-off between the coupling coefficient *q* and the entropy production σ for a fixed value of *x*, something pointed out by other authors [15,34].

### 3.3. Maximum Efficient Power Function

In this section, we present the Maximum Efficient Power regime given by Yilmaz et al. [6] which considers the effects on the design of heat engines, as the multiplication of power by the cycle efficiency, the criteria was successfully applied to the Carnot, Brayton, and Diesel engines, among other systems. From the above, the approach called maximum efficient power in the context of thermal engines is defined as,

(30) $$P_{e}=\eta P$$

where *P* is the power output. Maximization of this function provides a compromise between power and efficiency, where the designed parameters at maximum efficient power conditions lead to more efficient engines than those at the maximum power conditions [6].

In the context of the linear irreversible thermodynamics, the power efficient function takes the form,

(31) $$P_{e}=\frac{\Delta^{2}TA}{\left(1-\beta \right)}\left\{\frac{{\left[\frac{-[(1+\eta )\pm R}{2}\right]}^{2}{\left[\frac{-(1+\eta )\pm R)}{2}+1\right]}^{2}}{\left[\frac{-(1+\eta )\pm R)}{2}+{\left(1-\beta \right)}^{2}\right]}\right\}$$

when we take q=1 in Equation (31), we obtain:

(32) $$P_{e}=AT\Delta \eta^{2}\left[\Delta \left(1-\eta \right)\right]$$

Equations (31) and (32) have been obtained considering a new performance criterion, called efficient power, where its maximization leads to a compromise between power and efficiency. In the context of the Linear Irreversible Thermodynamics, the latter is interesting in the sense that we could compare not only the power output, but also the efficiency of the cycle.

### 3.4. Characteristic Functions vs efficiency

One point of interest in Linear Irreversible Thermodynamics is to obtain information about where the characteristic functions reach their maximum efficiency value; this can be found by means of $\frac{\partial F(\eta ,q)}{\partial \eta }$ where F(η,q) is any of the three cases considered (i.e., Maximum Power Output (MPO), Maximum Ecological Function (MEF) and Maximum Efficient Power Function (MEPF)).

For the Maximum Power Output (MPO) function, the efficiency which maximizes this function is given by;

(33) $$\eta_{MPO}=\frac{1}{2}\left(\frac{q^{2}}{2-q^{2}}\right)$$

**Figure 4:**
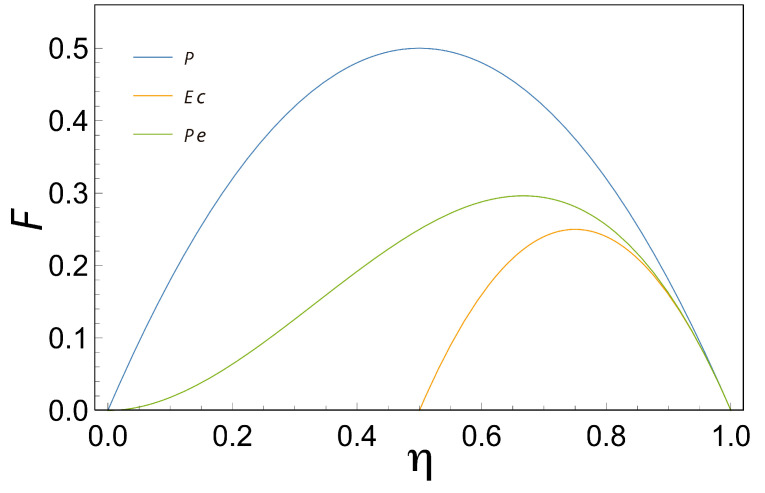
Characteristic Functions (P Maximum Power Output (MPO), Ec Maximum Ecological Function (MEF) and $P_{e}$ Maximum Efficient Power Function (MEPF)) as a function of the efficiency η. When we take q=1 we can see that Maximum Power Output reaches its maximum at η=0.5, the Ecological Function at η=0.75, and the Maximum Efficient Power Function at η=0.6666.

Note some interesting things about Equation (33); first, only when q=1, $\eta_{MPO}=\frac{1}{2}$ in the latest equation. The above is seen clearly in Figure 4 where Power Output (Equation (25)) has been plotted in terms of η and it is observed that the maximum is reached when η=1/2. Second, Equation (33) is the same as the one reported by [27], however, the result was obtained here by using a different approach. Third, if we perform a series expansion of Equation (32) in terms of *q* value around 0, we obtain: $\eta_{MPO}=\frac{q^{2}}{2}\left(\frac{1}{2}+\frac{q^{2}}{4}+O\left(q^{4}\right)\right)$ this last expression is in some sense equivalent to those founded for heat engines operating between two reservoirs [9,10,11,19].

Now, if we take the Ecological Function, and again we obtain the point where the efficiency maximizes the Ecological Function, we have;

(34) $$\eta_{MEF}=\frac{3}{4}\left(\frac{q^{2}}{4-3q^{2}}\right)$$

As in the MPO case, when q=1, $\eta_{MEF}=\frac{3}{4}$ in the latest equation, the above is observed clearly in Figure 4 where the Ecological Function (Equation (29)) has been plotted in terms of η and the maximum is reached when η=3/4 and q=1, besides Equation (34) is the same as the one reported by [27] but using a different approach. Moreover, performing an expansion of Equation (33) in terms of *q* we obtain; $\eta_{MEF}=\frac{q^{2}}{4}\left(\frac{3}{4}+\frac{9q^{2}}{16}+O\left(q^{4}\right)\right)$ and again, this last expansion is similar to those founded in [9,10,11,19].

Finally, for the case of the Maximum Efficient Power Function (MPEF) when q=1, $\eta_{MPEF}=\frac{2}{3}$. The above is shown clearly in Figure 4 where the Maximum Efficient Power Function (Equation (32)) has been plotted in terms of η and the maximum is reached when η=2/3. For that case, if we take the Efficient Power function, and we obtain the point where the efficiency maximizes the Maximum Efficient Power Function as a function of *q*, we obtain;

(35) $$\eta_{MPEF}=2*\left(\frac{4}{3q^{2}}-\frac{2}{3}-\frac{1}{12}\sqrt{-48+{\left(8-\frac{16}{q^{2}}\right)}^{2}}\right)$$

### 3.5. Loop-Shaped Curves

As Stucki pointed out [2] the coupling coefficient *q* in real biological systems usually is less than one, this could correspond to some sources of irreversibilities (for example, high thermal conductivity, among others) being different for each case depending on the system. What differs from one engine type to another is the magnitude and source of such irreversibility that gives rise to different power-efficiency curves of this shape [35]. The former has significant differences in the optimal operating conditions for real devices. Hence, looking for loop-shaped power-efficiency curves could aid us in studying the behavior mentioned above.

**Figure 5:**
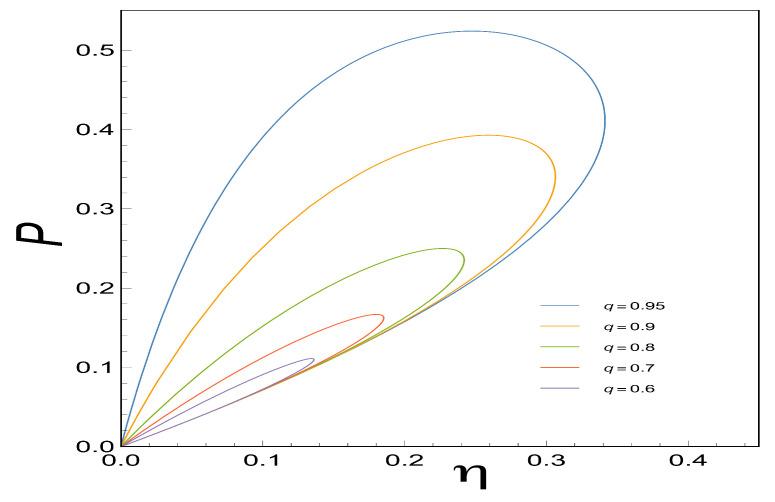
This Figure shows the Power Output *P* versus the Efficiency η for different values of *q*. We can observe how the power output produces loop-shaped curves as it is seen in real thermal engines.

**Figure 6:**
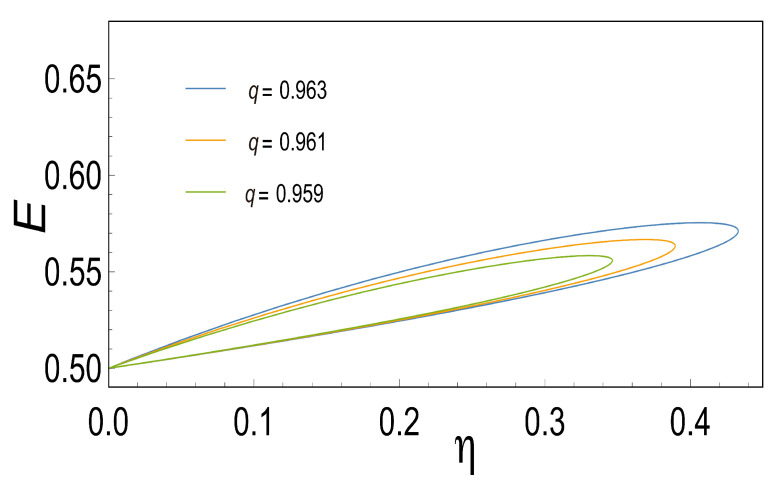
This Figure shows Ecological function *P* versus the Efficiency η for different values of *q*. We can observe how the Ecological function produces loop-shaped curves as it is seen in real thermal engines.

**Figure 7:**
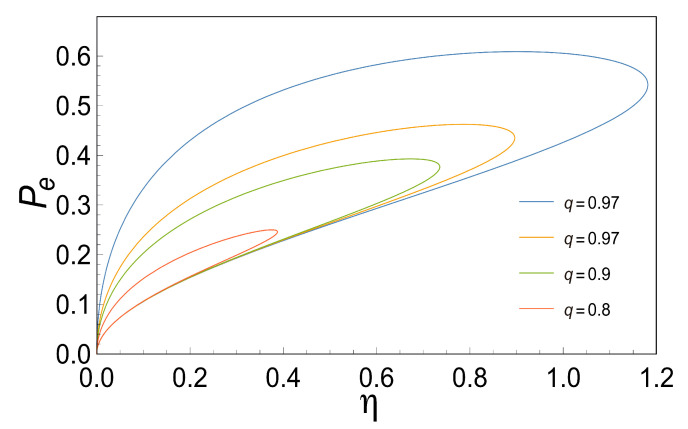
This Figure shows the Efficient Power Function $P_{e}$ versus the Efficiency η for different values of *q*. We can observe how the Efficient Power Function produces loop-shaped curves as it is seen in real thermal engines.

In order to gain information about the power-efficient curves for the case in which the coupling coefficient *q* is less than one, we proceed to analyze the loop-shaped curves using the functions of *P*, *E* and $P_{E}$ as a function of η. These are convex functions with respect to *x* (see Figure 3) when *q*=1. , when *q* is less than one, we observe that all of them describe a loop-shaped curve with some unusual characteristics such as the maximum obtained in each case for different values of *q*. The later shows how important the parameter *q* is since it reflects the behavior of the irreversibilities in the system (see Figure 5, Figure 6 and Figure 7).

## 4. Concluding Remarks

Many of the intra-cellular processes are studied based on some kind of a chemical reaction. In this work, by using a general model for enzymatic reaction that couples an exothermic with an endothermic reactions (and keeping in mind that most of the biochemical reactions in living organisms are catalyzed by enzymes), we analyzed the efficiency with which energy is exchanged between these reactions, but from the point of view of the non- equilibrium thermodynamics. By using the Linear Irreversible Thermodynamics, it is possible to analyze three different regimes of operation, namely, Maximum Power Output (MPO), Maximum Ecological Function (MEF) and Maximum Efficient Power Function (MEPF).

With that in mind, it is possible to obtain similar expressions for the Power Output and the Ecological Function previously reported by Diaz-Hernandez et al. [15], when the degree of coupling *q* is equal to one. It is worth mentioning that the studied model is completely based on well known biochemical facts, and in this work, in the context of the Linear Irreversible Thermodynamics, it is possible to generalize the obtained results, where all the thermodynamic features are determined by the chemical potential gap $\Delta =\frac{\mu_{1}-\mu_{4}}{RT}$, the efficiency η, and the degree of coupling *q*. Moreover, using the same formulation it is possible to add another regime named the Maximum Efficient Power Function in terms of the aforementioned parameters (Equation (31)).

Based on Figure 2, efficiency is a function of the force ratio $x=\frac{X_{1}}{X_{2}}$ for various values of *q*. Note the very rapid fall in $\eta_{max}$ with the decreasing of *q*. Again, in the limit q=1, we obtained results comparable to those obtained by Diaz-Hernandez et al. This limit has particularly important thermodynamic implications, since a perfect coupling implies that the flows are not linearly independent. Considering the above, we obtain the efficiency that maximizes the three characteristic functions (MPO, MEF, MPEF), when q=1, we observe that $\eta_{MPO}=0.5$, $\eta_{MEF}=0.75$ and $\eta_{MPO}=0.66$, as is shown in Figure 3.

However, in more realistic scenarios the coupling coefficient *q* is less than one, for instance Stucki [2] reported an experimental $q_{exp}=0.95$ for liver mitochondria in male rats. In this case (q<1), we have obtained the efficiency that maximizes the three characteristic functions and also the series expansions in terms of *q* resembling comparable results yielded by other already cited authors.

Since the three characteristic functions (see Equations (24), (28) and (31)) are determined by three parameters (Δ,q and η) and a variation of $\Delta =\mu_{1}-\mu_{4}$ (this can be achieved assuming a variation of the substrate and the end-product concentrations), the thermodynamic properties could improve because these characteristic functions are proportional to Δ. The latest could be the reason why some biomolecular machines can achieve high speed without sacrificing efficiency [36].

Now, from Equations (33), (34) and (35), it is clear that the efficiency that maximizes some of the characteristic functions is related only to *q*, so at the end, the thermodynamic properties are related to the degree of coupling providing the basis for comparing different types of coupling in a two-flow system. In other words, the relevant question in many biological situations could be: what is the efficiency with which free energy is exchanged between coupled chemical reactions? (question already made by other authors [26]). Here, the answer is in some sense clear; it depends only on the coupling coefficient and not on the individual phenomenological coefficients, which can be interpreted within the framework of non-equilibrium thermodynamics.
